# Rescue of Noradrenergic System as a Novel Pharmacological Strategy in the Treatment of Chronic Pain: Focus on Microglia Activation

**DOI:** 10.3389/fphar.2019.01024

**Published:** 2019-09-12

**Authors:** Filippo Caraci, Sara Merlo, Filippo Drago, Giuseppe Caruso, Carmela Parenti, Maria Angela Sortino

**Affiliations:** ^1^Department of Drug Sciences, Section of Pharmacology and Toxicology, University of Catania, Catania, Italy; ^2^Oasi Research Institute—IRCCS, Troina, Italy; ^3^Department of Biomedical and Biotechnological Sciences, Section of Pharmacology, University of Catania, Catania, Italy

**Keywords:** neuropathic pain, neuroinflammation, microglia, noradrenaline reuptake inhibitors, µ-opioid receptor agonists

## Abstract

Different types of pain can evolve toward a chronic condition characterized by hyperalgesia and allodynia, with an abnormal response to normal or even innocuous stimuli, respectively. A key role in endogenous analgesia is recognized to descending noradrenergic pathways that originate from the locus coeruleus and project to the dorsal horn of the spinal cord. Impairment of this system is associated with pain chronicization. More recently, activation of glial cells, in particular microglia, toward a pro-inflammatory state has also been implicated in the transition from acute to chronic pain. Both α2- and β2-adrenergic receptors are expressed in microglia, and their activation leads to acquisition of an anti-inflammatory phenotype. This review analyses in more detail the interconnection between descending noradrenergic system and neuroinflammation, focusing on drugs that, by rescuing the noradrenergic control, exert also an anti-inflammatory effect, ultimately leading to analgesia. More specifically, the potential efficacy in the treatment of neuropathic pain of different drugs will be analyzed. On one side, drugs acting as inhibitors of the reuptake of serotonin and noradrenaline, such as duloxetine and venlafaxine, and on the other, tapentadol, inhibitor of the reuptake of noradrenaline, and agonist of the µ-opioid receptor.

## Introduction

Chronic pain, a disease entity with a major impact on healthcare costs, is characterized by hyperalgesia, an increased response to noxious thermal and mechanical stimuli, and allodynia, in which nociceptive responses occur to normally innocuous stimuli such as light touch (known as mechanical allodynia). Pain of different origins including: (i) inflammatory pain following tissue injury, (ii) cancer pain, and (iii) neuropathic pain following nerve, spinal cord or brain (e.g., stroke) injuries (Costigan et al., 2009; Ji et al., 2013; Finnerup et al., 2015) can become chronic.

Chronic “pathologic” pain results from a maladaptive functional and structural transformation process, sustained by mechanisms of peripheral and central sensitizations involving an altered neuronal activity. These include sensitization of peripheral primary sensory neurons in the dorsal root ganglia and trigeminal ganglia as well as sensitization of central nociceptive neurons in the spinal cord, trigeminal nucleus, brain stem, and cortex. These events have been deeply investigated at molecular and cellular level in animal models of acute and chronic pain (Scoto et al., 2009; Ji et al., 2014; Cairns et al., 2015; Coluzzi et al., 2017), and a key role in the transition from acute to chronic pain has been recognized to glial cells, both astrocytes and microglia (Cao and Zhang, 2008; Ji et al., 2014). Drug discovery efforts for the therapy of chronic pain have been recently directed to develop “context-dependent drugs” able to exert analgesic effects in an early phase of pain chronicization (Vicario et al., 2019). Focusing on treatments that target key elements in the pathophysiology of chronic pain will improve the chances to develop therapies that go beyond current symptomatic treatment. Indeed, identification of new molecular targets involved in the pathogenesis of chronic pain represents an essential step for the design of disease-modifying analgesic drugs able to counteract the progression of this disease and, most importantly, to prevent its transition to a chronic state.

In the present review, we will briefly examine two key elements in the pathophysiology of chronic pain, recently proven to be correlated, i.e., the noradrenergic system and neuroinflammation. We will then discuss drugs that act by rescuing the noradrenergic system as a new potential pharmacological strategy for the treatment of chronic pain.

## The Impairment of Noradrenergic System in the Transition From Acute to Chronic Pain

Descending monoaminergic inhibitory pathways project from the brain stem to the spinal cord and finely regulate pain threshold (Millan, 2002). These include serotonergic fibers originating from the raphe nuclei and noradrenergic descending fibers originating from the locus coeruleus (Llorca-Torralba et al., 2016). Activation of descending serotonergic pathways has been shown to play a central role in the analgesic effects of tricyclic antidepressants, selective serotonin reuptake inhibitors (SSRIs), and serotonin–noradrenaline reuptake inhibitors (SNRIs e.g., duloxetine and venlafaxine) in acute models of pain (Zammataro et al., 2017). However, recent studies suggest that the serotoninergic system can exert an ambivalent role in pain transmission. Activation of different serotonin receptors can in fact produce either pro-nociceptive effects (5-HT2A, 5-HT3 receptors) or anti-nociceptive effects (5-HT1A, 5-HT7 receptors) (Bardin, 2011). On the contrary, descending noradrenergic pathways exert a more prominent role under conditions of persistent pain, producing an inhibitory effect on pain transmission (Caputi et al., 2019; Hayashida and Obata, 2019). This has been reported in acute, inflammatory, and neuropathic pain models (Pertovaara, 2013). Accordingly, the analgesic effect of duloxetine strongly derives from the activation of descending noradrenergic pathways (Zhao et al., 2007; Kremer et al., 2018).

The largest noradrenergic nucleus, the locus coeruleus, is located in the dorsal pons and contains more than 50% of all noradrenergic neurons (Singewald and Philippu, 1998). Several preclinical studies have demonstrated that the descending noradrenergic pathways from the ventral locus coeruleus (i.e., large multipolar neurons), projecting to the dorsal horn of the spinal cord, reduce spinal pain transmission exerting a prominent role in endogenous analgesia (Millan, 2002; Llorca-Torralba et al., 2016; Patel et al., 2018). Multiple mechanisms are implicated in the anti-nociceptive effects of noradrenaline in the spinal dorsal horn, and they involve both neurons (Hayashida and Obata, 2019) and glial cells (Arora et al., 2016). Noradrenaline stimulates both pre- and postsynaptic α2-adrenergic receptors coupled to inhibitory G protein (Gi/o) (Pertovaara, 2013). Stimulation of presynaptic α2-adrenergic receptors on primary nociceptive neurons inhibits voltage-gated Ca^2+^ channels, thus reducing the release of excitatory neurotransmitters (i.e., glutamate and substance P) (Pertovaara, 2013). On the other hand, activation of postsynaptic α2-adrenergic receptors on secondary sensory neurons in the spinal cord results in their hyperpolarization through the opening of inwardly rectifying K^+^ channels, with ensuing reduction of neuronal excitability (Hayashida and Obata, 2019).

The endogenous noradrenergic analgesic system plays a key role in shaping the spatial and temporal expression of the neuropathic pain phenotype after nerve injury (Hughes et al., 2013). Studies in animal models have demonstrated that in a relatively early stage of neuropathic pain following peripheral nerve injury, descending noradrenergic pathways exert an effective inhibition against mechanical and thermal hypersensitivity by increasing brain-derived neurotrophic factor (BDNF) levels (Hayashida et al., 2008; Hayashida and Eisenach, 2010). These molecular events are also associated with an increased expression and activity of pre- and postsynaptic α2-adrenoceptors to attenuate glutamatergic transmission in the spinal dorsal horn (Chen et al., 2011).

Impairment of endogenous adrenergic analgesia is thought to be responsible for the transition from acute to chronic pain (**Table 1**). Indeed, in later phases of neuropathic pain, noradrenergic neurons in the locus coeruleus become less responsive to noxious stimuli, due to a dysfunction in the glutamatergic system that controls noradrenaline release (Kimura et al., 2015). Accordingly, the selective deficiency of descending inhibitory modulation promotes the transition from acute to chronic pain in neuropathic rats (Patel et al., 2018). In addition, the noradrenaline transporter (NET) was found to be up-regulated in the spinal cord of a neuropathic rat model (Rojo et al., 2012), highlighting, again, the significance of descending noradrenergic pathways in endogenous analgesia. Interestingly, the translational potential of these preclinical studies is supported by observations in patients with neuropathic pain who show a reduced ability to recruit descending inhibition (Lewis et al., 2012). The relevance of the descending noradrenergic system in pain chronicity is demonstrated by the development of conditioned pain modulation (CPM) as a diagnostic tool in quantitative sensory testing (Yarnitsky et al., 2012). CPM is a paradigm whereby heterotopic noxious stimulation reduces pain perception of a test stimulus, providing a readout of the integrity of net endogenous inhibitory descending pathways. Patients with low CPM show an increased propensity to develop chronic pain after surgery. Interestingly, patients with neuropathic pain and low CPM show a high rate of response to treatment with duloxetine or with tapentadol (a multimodal analgesic which acts as µ-opioid receptor agonist [MOR] and noradrenaline reuptake inhibitor) (Yarnitsky et al., 2012). Given the importance of the deficits of noradrenergic system early during pain chronicization, we believe that the mechanisms involved should be reconsidered not only from a “neuronal” perspective, but also taking into account the recent evidence on the key role of neuroinflammation in the transition from acute to chronic pain, which suggests a protective role for noradrenaline also against pro-inflammatory glial activation.

**Table 1:** Impairment of descending noradrenergic system in neuropathic pain: evidence from animal models.

Animal model	Preclinical phenotype	Reference
Spinal nerve ligation	Enhanced stimulus-evoked and spontaneous firing reduced by clonidine	Patel et al., 2018
L5-L6 spinal nerve ligation	Increased extracellular glutamate in the LC and impaired pain-evoked endogenous analgesia after nerve injury	Kimura et al., 2015
L5-L6 spinal nerve ligation	Mechanical hypersensitivity reduced by α2-agonists	Hayashida et al., 2008
Streptozotocin-induced diabetic rats	Mechanical allodynia and thermal hyperalgesia reduced by duloxetine	Kinoshita et al., 2013
Rats with tibial nerve transection	Mechanical and cold allodynia and heat hypersensitivity, all increased by α2-antagonists	Hughes et al., 2013
Incisional pain model combined with DβH-saporin	Selective degeneration of NA neurons with delayed recovery of mechanical hypersensitivity and increased spinal glial activation reduced by α2-agonists	Arora et al., 2016

LC, locus coeruleus; DβH-saporin, dopamine β-hydroxylase conjugated to saporin; NA, noradrenaline.

## Neuroinflammation and Microglial Activation in the Pathophysiology of Chronic Pain: A Protective Role for Noradrenaline

Neuroinflammation is characterized by infiltration of immune cells, glial activation, and production of inflammatory mediators in the peripheral and central nervous system (CNS), and it plays a central role in the pathophysiology of chronic pain (Ji et al., 2014; Lees et al., 2015). During the transition from acute to chronic pain, peripheral damage and hyperactivity of primary sensory neurons promote neuroinflammation through the release of several pro-inflammatory cytokines (e.g., TNF-α and IL-1β), chemokines, glutamate, and reactive oxygen species by both activated astrocytes and microglial cells (Lees et al., 2015; Vicario et al., 2016). These glial mediators modulate excitatory and inhibitory synaptic transmission in the spinal cord by enhancing long-term potentiation and nociceptive neurotransmission, finally leading to central sensitization and pain chronicization (Ji et al., 2014). Glial activation, involving both astrocytes and microglia, represents a common pathophysiological event in chronic pain, Alzheimer's disease (AD), and also depression, although neurodegenerative disorders are characterized by significant neuronal loss (Caraci et al., 2018).

Microglia are the resident immune cells of the CNS, with a primary role in maintaining CNS homeostasis, but are rapidly activated in response to any subtle change in the surrounding microenvironment (Wolf et al., 2017). Based on this dual activity and state of activation, microglia phenotypes have been simplified as “M1” and “M2” (Cherry et al., 2014). The M1 state represents a reactive phenotype releasing several pro-inflammatory molecules, physiologically involved in host defense, but also in pathological neuroinflammation (Du et al., 2017; Kabba et al., 2018). On the contrary, M2 represents a surveillance mode whereby microglia constantly monitor the environment and are involved in neurodevelopmental and restorative processes (Kabba et al., 2018). Nevertheless, it has become increasingly clear that microglia actually display a wide range of intermediate phenotypes in a continuum between M1 and M2 (Ransohoff, 2016).

In the context of pain, polarization toward the M1 phenotype occurs following tissue injury and stress and is accompanied by the release of several inflammatory cytokines (IL-1β, IL-6, TNF-α). On the other hand, M2 polarization significantly contributes to resolution of inflammation and tissue repair, through phagocytic activity and release of anti-inflammatory cytokines such as IL-4, IL-10, and TGF-β1, which antagonize central sensitization and pain chronicization (Milligan et al., 2006; Lantero et al., 2012; Grace et al., 2014). Studies in animal models of neuropathic pain have demonstrated that activated spinal microglia (Tsuda et al., 2008) release pro-inflammatory cytokines (IL-1β, IL-6, TNF-α) and lead to phosphorylation of the mitogen-activated protein kinases (MAPKs) including p38-MAPK, extracellular signal-regulated protein kinase (ERK), and c-Jun N-terminal kinase (JNK), which are known to participate in central sensitization and generation of pain hypersensitivity (Obata and Noguchi, 2004; Wang et al., 2014). A recently emerging approach in drug discovery, for diseases characterized by underlying neuroinflammation, is therefore the modulation of microglial polarization and selective regulation of the release of pro-/anti-inflammatory molecules (Haight et al., 2019). This could represent a promising new pharmacological strategy for treatment of chronic pain but also of other disorders such as chronic pain-associated affective disorders and depression (Benatti et al., 2016; Pena-Altamira et al., 2016; Du et al., 2017; Barcelon et al., 2019).

Although microglia appear to drive neuroinflammatory mechanisms in pain chronicization, an important role in central sensitization and chronic pain is also played by spinal astrocytes (Chen et al., 2019). The time course of astrocyte reactivity appears to be delayed compared to that of microglia (Gwak et al., 2012), but astrocyte activation can in some conditions be persistent and correlated with chronic pain states (Liao et al., 2011; Ji et al., 2014).

Which is the role of noradrenergic system in this scenario? Can noradrenaline prevent glial cells activation, and can it exert a protective and anti-inflammatory role in the context of neuroinflammation related to chronic pain?

Noradrenaline is known to exert strong anti-inflammatory activity in the CNS (Mcnamee et al., 2010) and its endogenous neuroprotective role in chronic pain is likely linked to this action (Zhang et al., 2016b).

As mentioned, noradrenaline, released from descending bulbospinal neurons in the spinal dorsal horn, decreases nociceptive transduction through the activation of neuronal α2-adrenergic receptors, at both pre- and post-synaptic levels (Pertovaara, 2013) (see above). In addition, it suppresses pain transduction *via* activation of GABAergic and glycinergic inhibitory interneurons (Baba et al., 2000). α2-Adrenoceptors are expressed on both astrocytes and microglial cells in the spinal cord (Mori et al., 2002; Morioka et al., 2014), and several studies have demonstrated that their stimulation reduces glial activation after peripheral nerve injury or during chronic inflammation (Xu et al., 2010). It has been hypothesized that spinally projecting noradrenergic pathways and activation of spinal α2-adrenergic receptors are important for speeding recovery from hypersensitivity occurring after surgical incision, an effect likely linked, also in this case, to reduction of spinal glia activation (Arora et al., 2016). Accordingly, in spinal cord glia of an animal model of neuropathic pain, the α2-adrenergic receptor agonist clonidine inhibits inflammatory markers such as nuclear factor-kappa B (NF-kappaB) and p38 MAPK and the release of pro-inflammatory cytokines (IL-1β, IL-6) (Feng et al., 2009). Interestingly, clonidine has also been shown to reduce hypersensitivity after L5–L6 spinal nerve ligation, and this effect involves sprouting of noradrenergic fibers in the spinal cord and enhanced expression of α2-adrenergic receptor (Hayashida et al., 2008). This action requires BDNF, presumably of microglial origin (Coull et al., 2005), suggesting an additional role for microglia in modulating descending noradrenergic pathways in the control of chronic pain. Moreover, chronic analgesic effects of tramadol on neuropathic pain induced in rats by partial sciatic nerve ligation have been ascribed to α2-adrenoceptor-mediated inhibition of astrocytic activation (Sakakiyama et al., 2014). The involvement of an anti-inflammatory action of noradrenergic pathways in pain relief was further confirmed by evidence that a selective disruption of descending spinal noradrenergic fibers was associated with: (i) delayed recovery of mechanical hypersensitivity and (ii) enhanced expression of both microglial (Iba1) and astrocytic (GFAP) markers in the ipsilateral spinal cord, 21 days post-incision (Arora et al., 2016).

β-Adrenergic receptors are also abundantly expressed in glial cells of the rat spinal dorsal horn (Mizukami, 2004; Nicholson et al., 2005), where their activation was shown to control nociceptive transduction by attenuating microglial reactivity (Morioka et al., 2009; Zhang et al., 2016b). Systematic treatment with β-adrenergic receptor agonists resulted in antinociceptive effects in animal models of neuropathic pain (Choucair-Jaafar et al., 2009). Stimulation of spinal dorsal horn β2-adrenergic receptors in mice was also shown to ameliorate neuropathic mechanical hypersensitivity, following a partial sciatic nerve ligation, through reduction of phosphorylation of microglial p38 MAPK and astrocytic JNK (Zhang et al., 2016b). Evidence obtained in animal models is supported by *in vitro* studies in cultured rat spinal microglia where noradrenaline, *via* β-adrenergic receptors, downregulates inflammatory signaling including ATP-induced cAMP–protein kinase A–dependent phosphorylation of p38 MAPK and synthesis of TNF-α (Morioka et al., 2009). Further studies are however needed in animal models to validate the role of glial β2-adrenergic receptors as a novel pharmacological target for the treatment of chronic pain.

**Figure 1:**
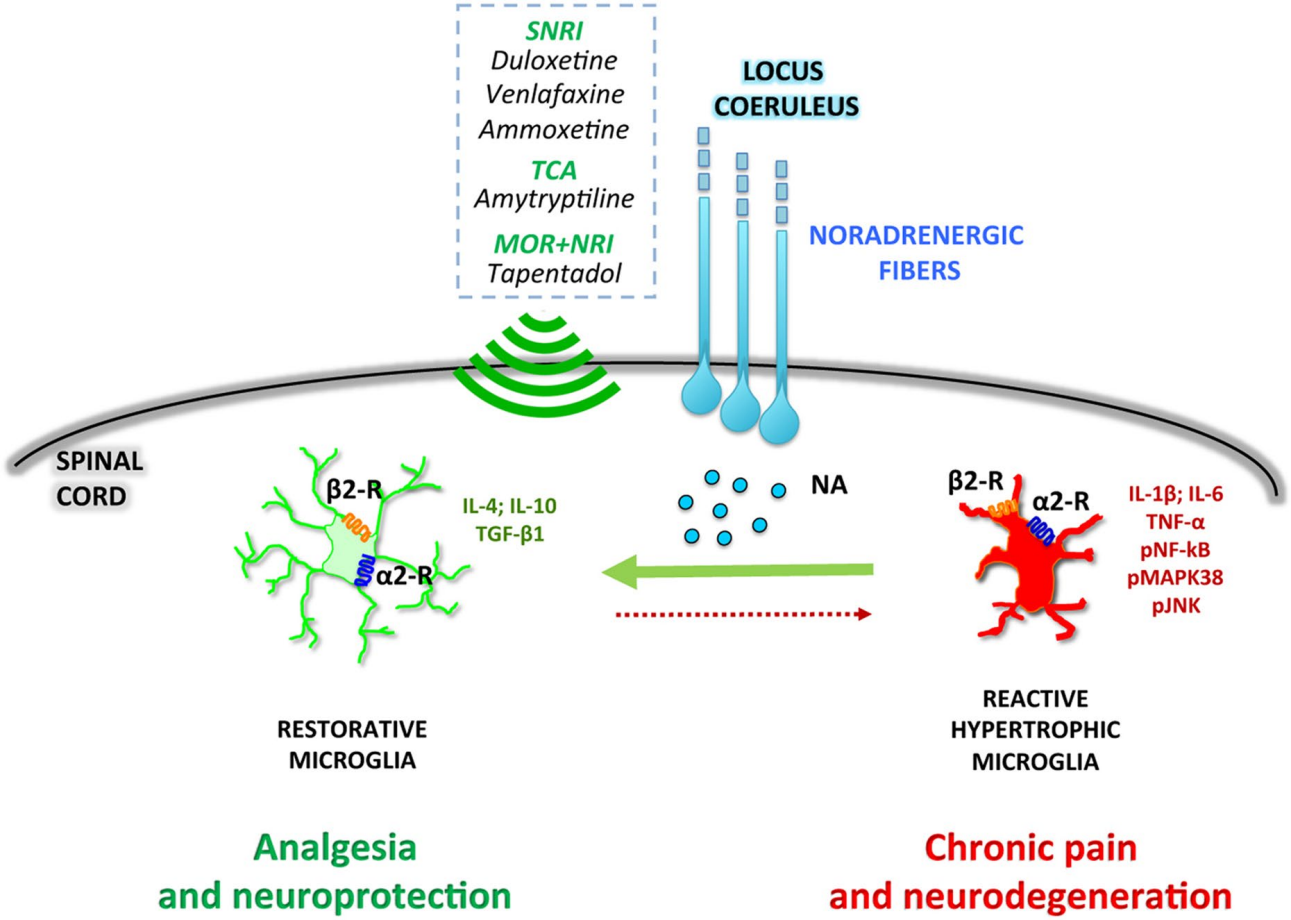
Noradrenergic fibers projecting from the locus coeruleus release noradrenaline (NA) in the dorsal horn of the spinal cord. NA directly modulates microglial polarization through both α2- and β2-receptors (α2-R and β2-R), promoting a shift toward a restorative, anti-inflammatory phenotype. Drugs activating the noradrenergic system, such as serotonin–noradrenaline reuptake inhibitors (SNRI), tricyclic antidepressants (TCA), or combined μ-opioid receptor agonists and noradrenaline reuptake inhibitors (MOR+NRI) potentiate the microglial anti-inflammatory phenotype, resulting in analgesic and neuroprotective effects.

Overall, the preclinical data here reported suggest that the selective deficiency of noradrenergic system impacts both at neuronal and glial levels. As said, in an early phase of chronic pain pathophysiology, peripheral nerve injury enhances endogenous spinal noradrenergic tone which negatively modulates glial activation and hypersensitivity. However, this early endogenous high noradrenergic tone might not be by itself sufficient to relieve neuropathic pain (Hayashida et al., 2008) and may be reduced in a later phase with progression of pain chronicization (Kimura et al., 2015). According to this scenario, the rescue of the noradrenergic system might represent a novel pharmacological approach to prevent the transition from acute to chronic pain (**Figure 1**).

## Rescue of Noradrenergic System as a Novel Pharmacological Approach to Reduce Microglia Activation and Neuroinflammation in Chronic Pain

Targeting activated microglia with drugs able to rescue the noradrenergic system has recently emerged as a novel approach to prevent the transition from acute to chronic pain (Yamashita et al., 2016; Kremer et al., 2018; Tawfik et al., 2018; Zhang et al., 2018). Preliminary evidence obtained with the SNRIs duloxetine and ammoxetine opens the path for future studies with analgesic drugs, such as tapentadol, which combines MOR activation and potentiation of noradrenergic system.

Duloxetine is known to prevent pain hypersensitivity in models of neuropathic pain associated with traumatic nerve injury (Iyengar et al., 2004; Le Cudennec and Castagne, 2014; Hoshino et al., 2015). Similarly, ammoxetine, a novel and potent SNRI, exhibited a strong analgesic effect in models of inflammatory, neuropathic and fibromyalgia-related pain (Zhang et al., 2016a; Zhang et al., 2018). Inhibition of microglial activation has been recently identified as a new mechanism which strongly contributes to the analgesic effects of duloxetine (Kremer et al., 2018; Tawfik et al., 2018). The first evidence in this regard comes from studies carried out in animal models of chemotherapy-induced neuropathy (Greish et al., 2014) and intervertebral disc-related neuropathic pain (Handa et al., 2016). Furthermore, the inhibition of mechanical allodynia and thermal hyperalgesia induced by duloxetine, in a mouse model of diabetic neuropathy, was paralleled by a significant reduction of specific markers of microglia (CD11b) and astrocyte (GFAP) activation (Tawfik et al., 2018). Interestingly, in this animal model, duloxetine also rescued nerve growth factor (NGF) mRNA levels, which were reduced in the sciatic nerve. This effect was confirmed in chemotherapy-induced peripheral neuropathy in which duloxetine inhibited the inflammatory response, by reducing p38 MAPK phosphorylation and NF-κB nuclear translocation, and controlled nerve degeneration by rescuing NGF levels (Meng et al., 2019).

Different molecular mechanisms seem to contribute to the effect of duloxetine on microglia. The antiallodynic effect of duloxetine in rats is in fact significantly reduced, but not abolished, after pretreatment with N-(2-chloroethyl)-N-ethyl-2-bromobenzylamine (DSP-4), a neurotoxin selective for noradrenergic neurons (Yamashita et al., 2016). Duloxetine exerts also an inhibitory effect on the function of P2X4 purinergic receptors, a subtype of ATP-gated non-selective cation channels, highly upregulated in spinal microglia after peripheral nerve injury (Yamashita et al., 2016). This may represent a new potential therapeutic target for the treatment of neuropathic pain. Recently, Kremer et al. (2018) reported that duloxetine and the tricyclic antidepressant amitriptyline exert a dual response in a model of neuropathic pain induced by sciatic nerve compression. An acute anti-allodynic action, secondary to activation of central α_2A-_ adrenoceptors, MOR, and δ-opioid receptors (DOR), and a delayed analgesic effect mediated by activation of peripheral β_2_ adrenoceptors and DOR, leading to inhibition of the TNF-α–NF-κB pathway (Kremer et al., 2018). Of note, this late, peripheral component of duloxetine action is related to inhibition of neuroimmune mechanisms that accompany nerve injury. The involvement of β_2_ adrenoceptors and the inhibition of TNF-α signaling is also a key mechanism for the analgesic action of the noradrenergic antidepressant nortriptyline, as observed in animal models of neuropathic pain (Bohren et al., 2013).

The novel SNRI ammoxetine inhibits microglia activation, as shown by inhibition of LPS-induced Iba-1 expression in the BV-2 microglial cell line. The inhibition of the inflammatory response has been suggested to be at the basis of its analgesic activity. In streptozocin-induced diabetic rats, chronic treatment with ammoxetine relieved mechanical allodynia and reversed depressive-like phenotype by inhibiting, in spinal microglia, both p38 MAPK and JNK signaling pathways (Zhang et al., 2018). Of note, in this model of neuropathic pain, there was marked activation of microglia, suggested also by strong induction of Iba-1, but no evidence of astrocytic activation (Zhang et al., 2018). In contrast, other studies using the same animal model have detected strong activation of microglia and astrocytes at different times after the appearance of neuropathy (Cheng et al., 2014).

On these bases, it can be hypothesized that drugs that are able to rescue the noradrenergic system, such as SNRIs and tapentadol, can exert their analgesic efficacy by inhibiting microglia activation, thereby preventing the transition from acute to chronic pain. Among microglial anti-inflammatory cytokines, TGF-β1 has protective effects against the development of chronic neuropathic pain by inhibiting neuroinflammation and promoting the expression of endogenous opioids within the spinal cord (Lantero et al., 2012). Interestingly, therapeutic concentrations of the SNRI venlafaxine prevent microglial activation reduce pro-inflammatory cytokine secretion and increase the release of TGF-β1, as reported in an astroglia–microglia co-culture (Vollmar et al., 2008). As discussed above, central sensitization and maladaptive plasticity play a central role in the pathophysiology of chronic pain. We have recently identified a key role for TGF-β1 in synaptic plasticity and in the transition from early to late LTP (Caraci et al., 2015). In addition, a TGF-β1-opioid receptor signaling crosstalk results in improvement of endogenous and exogenous opioid analgesia in experimental models of neuropathic pain (Lantero et al., 2012). It is unknown whether tapentadol, which combines activation of MOR with the inhibition of noradrenaline reuptake (Raffa et al., 2018), can positively modulate TGF-β1 signaling in microglial cells by rescue of noradrenergic system.

Tapentadol is the only approved centrally acting analgesic that was developed from the beginning to enhance analgesic efficacy by combining two specific synergistic mechanisms of analgesic action (Raffa et al., 2018). Recent studies demonstrate the clinical efficacy of tapentadol in a broad spectrum of acute and chronic pain conditions including post-surgical, musculoskeletal, and neuropathic pains (Langford et al., 2016). The analgesic efficacy of tapentadol is only partially derived from opioid-mediated mechanisms. Recent findings indicate that the µ-load of tapentadol is low (40%) when compared to pure MOR agonists (i.e., the % contribution of the opioid component to the adverse effect magnitude relative to a pure/classical µ-opioid at equianalgesia) (Raffa et al., 2018). This reduced µ-load is relevant not only to explain the improved tolerability profile of tapentadol and the reduced incidence of some of the typical opioid-induced side effects (Langford et al., 2016) but also, most importantly, to reconsider noradrenergic system rescue as a key mechanism responsible for the strong analgesic effects of tapentadol in chronic pain. Future studies both in microglial cells and in animal models of neuropathic pain will be essential to understand whether rescue of noradrenergic system and associated inhibition of microglia activation contribute to the overall analgesic efficacy of tapentadol, representing a novel key mechanism to prevent the transition from acute to chronic pain.

## Author Contributions

All authors gave substantial contributions to the conception and design of the review and approved the final version.

## Funding

The authors declare that the review was conducted with the unrestricted support of Grunenthal. In this review the funders had no role in review design, data collection and analysis, decision to publish, or preparation of the manuscript.

## Conflict of Interest Statement

The authors declare that the research was conducted in the absence of any commercial or financial relationships that could be construed as a potential conflict of interest.
